# Internet Use, Life Satisfaction, and Subjective Well-Being Among the Elderly: Evidence From 2017 China General Social Survey

**DOI:** 10.3389/fpubh.2021.677643

**Published:** 2021-06-29

**Authors:** Hua-lei Yang, Yuan-yang Wu, Xue-yu Lin, Lin Xie, Shuo Zhang, Si-qing Zhang, Shi-ming Ti, Xiao-dong Zheng

**Affiliations:** ^1^School of Public Administration, Zhongnan University of Economics and Law, Wuhan, China; ^2^Institute of Population and Labor Economics, The Chinese Academy of Social Science, Beijing, China; ^3^School of Economics, Zhejiang Gongshang University, Hangzhou, China

**Keywords:** internet use, life satisfaction, elderly, subjective welfare, China

## Abstract

**Purpose:** The research objectives of this study are to test the scientific propositions of whether Internet use promotes life satisfaction among the elderly, whether there is variability in the effect of Internet use on the well-being of the elderly, and through what channels Internet use affects the elderly's life satisfaction.

**Method:** Using data from the 2017 Chinese General Social Survey (CGSS), this paper employed linear regression, ordered logit model, and the propensity score matching (PSM) approach to investigate the association of Internet use with life satisfaction among the elderly in China.

**Results:** The results show that Internet use was significantly and negatively associated with the life satisfaction of the Chinese elderly. Further analysis on group heterogeneity demonstrates that the negative association was more prominent among the participants who were males, at a lower age, had lower income and education, non-agricultural registered, and relatively healthy. Finally, the mediating effect indicates that Internet use may affect life satisfaction among the elderly through the channel of reducing their perceptions of social justice.

**Conclusions:** In order to avoid the negative effects of using the Internet, the following policy suggestions are put forward: Improve the elderly's attitudes toward Internet use; pay attention to the sense of fairness of the elderly to improve life satisfaction; guide the elderly to reduce the time of using the Internet.

## Introduction

In the context of the increasingly severe world aging situation, how to deal with aging is one of the major challenges facing the world and China today. Since the 1990s, the Internet and other information technologies have developed rapidly in the world and China. In June 2020, the number of Internet users in China had reached 940 million, and the Internet penetration rate has reached 67.0%. At the same time, the proportion of elderly Internet users are also increasing. The proportion of Internet users aged 60 and above has increased from 6.7% in March to 10.3% in June. It has become an essential part of people's daily lives. With the rapid development of the Internet, how the elderly, as the most respectable and caring group in the whole society, can be made to feel more happier in the use of the Internet has attracted wide attention from the academic community.

Psychological well-being (PWB) and subjective well-being (SWB) are the two directions of contemporary psychological research on well-being. The main difference between them lies in the different definitions of happiness. SWB is an overall assessment of the quality of people's lives based on internal criteria, a comprehensive evaluation of people's satisfaction with life and its various aspects, and the resulting psychological state dominated by positive emotions (1). SWB consists of three main aspects: life satisfaction, positive emotions, and negative emotions. The higher the overall satisfaction with life, the more positive emotions experienced, and the less negative emotions experienced, the greater the person's well-being (2). PWB mainly refers to a good state of human psychological functioning, a perfect realization of self-improvement, self-actualization, and self-achievement, a perfect realization of self-potential that does not depend on one's subjective will (3). PWB includes six aspects: self-acceptance, positive relationships with others, functional autonomy, environmental control, life goals, and personal growth (4). Among the concepts used in the current study, SWB, well-being, and PWB are basically close in meaning and are all based on the conceptual model of SWB. The study of SWB, as an important indicator of the quality of life of the elderly, can help improve the quality of life of the elderly. In addition, traditional studies on SWB have focused on the effects of income, education, public expenditure, government quality, and social capital on residents' SWB, while less attention has been paid to the role of technology applications, especially information technology (5). Therefore, this paper will explore the relationship between Internet use and SWB of the elderly.

Life satisfaction is an important indicator reflecting the level of SWB of the elderly. It reflects people's subjective evaluation of the individual's comprehensive quality of life (6). Many studies have shown that lower life satisfaction is highly associated with mental illness (7), depressive symptoms (8), and suicidal ideation (9). Therefore, this study chose “life satisfaction” as the core indicator to measure the impact of the Internet use on the lives of the elderly. The impact of the Internet use on the life satisfaction of the elderly is multiple, so this paper will review the current literature on the impact of Internet use on people's life satisfaction in terms of both positive and negative effects.

On the one hand, the Internet use may enhance the life satisfaction of older adults. First, the Internet can provide more access to information, promote social participation, and further enhance their life experience and happiness. The Internet also effectively reduced the cost of residents' access to information (10), which leads to better entertainment and online consumption (11). Openness, anonymity, virtuality, and equality of the Internet encouraged the elderly to participate in current affairs, which can improve their self-efficacy and reduce their stress and depression levels (12–14). Using the Internet, the elderly could better participate in the community; expand the social network; and improve self-rated health, mental health, and social adaptation levels, thereby improving the overall life satisfaction of the elderly (15–19). For example, Opalinski (20) used 110 observations of elderly over 60 years old in the United States, Canada, and other countries, and found that social activities or learning through the Internet can increase life experience and reduce their loneliness. Shapira et al. (21) used 48 elderly living in nursing homes as experimental subjects and found that learning to use the Internet may benefit their life experience by improving cognitive ability and self-care ability.

Second, Internet could enhance the elderly's connection with the outside world, expand the scope of communication, increase social support, avoid social isolation, and have a significant happiness incentive effect on the elderly (15, 22–27). The primary purpose of most elderly using the Internet is to send and receive emails and communicate effectively with others. The elderly maintained close contact with their children, relatives, and friends through the Internet; helped them overcome time and space barriers; and increased their face-to-face interaction time, thereby enhancing their happiness (28–31). For instance, Chopik (32) took 591 elderly as the research object and found that the elderly could reduce loneliness and the probability of depression using email, social networking sites, and online chat. McMellon and Schiffman (33), Nahm and Resnick (34), O'Hara (35), and Winstead et al. (36) found that the Internet is beneficial for interpersonal communication and maintaining family bonds. For the elderly with withdrawn personalities and low social participation, the Internet could increase their connection and support with social networks, thereby improving happiness to a certain extent (37). Heo et al. (38), based on the 2008 US HRS (Health and Retirement Study) data, used structural equation models and found that the Internet reduces loneliness by improving social support as the intermediary variable for the elderly. On the other hand, Internet use may reduce life satisfaction of older adults. First, addiction to the Internet will reduce communication in real life. Excessive time spent on weak ties on the Internet would reduce the time spent on strong ties, which will weaken the relationships in the real life and have a negative impact on life satisfaction. The “Internet-time substitution hypothesis” proposes that residents' use of the Internet will occupy time for social interaction, resulting in reduced communication with family members, reduced opportunities for face-to-face communication between people, and increased isolation between people. It is harmful to maintaining personal and social relationships, thereby increasing inner loneliness and reducing life satisfaction (39–41). Gilleard et al. (42) and Hage et al. (43) believed that using the Internet could cause the elderly to reduce their valid social participation and weaken the elderly's sense of belonging to the community, thereby causing a negative impact on the elderly's life satisfaction. Matsuba (44), Stepanikova et al. (45), Odaci and Elik (46), Azher et al. (47), Noh and Kim (48), Sami et al. (49), and Kitazawa et al. (50) found that excessive use of the Internet could make people addicted, produce loneliness, and affect mental health and life satisfaction.

Second, Internet use will expand the range of comparison. Relative income level and social status play a decisive role in a person's happiness (51–54). The social comparison of Internet use will affect people's happiness experience. According to the theory of reference groups, people's cognition and judgment of their living conditions mostly do not depend on their current social environment status but by comparing with other reference objects. Based on the theory of interpersonal interaction, the generation of an individual's SWB results from comparing one's own life with others. Social Comparison Theory (SCT) points out that the level of SWB is often the result of social comparison (55, 56), When compared with individuals who are better than yourself, it would harm the individual and reduce happiness (57). The popularity of the Internet would increase access to information for the elderly and increase people's expectations of material needs, making it easier for them to compare with others online from any country with any background. Comparing behavior based on this would bring about psychological loss and relative deprivation, thereby reducing people's happiness in life (58–62).

From the above literature review, we can see that there are following points to improve on: (1) In terms of research objects, affected by factors such as the low level of economic development and the late start of the Internet industry, there is few of the literature research around Chinese elderly; (2) the volume of research conducted based on the latest 2017 China General Social Survey (CGSS) is almost empty in terms of sample selection. The research conclusions based on China's situation need to be further verified; (3) in terms of research content, the depth of analysis of heterogeneity based on age group, education, health, and income is not sufficient; (4) in terms of research methods, the endogenous problem caused by self-selection is frequently neglected.

This article starts from the perspective of the life satisfaction of the elderly; draws on relevant classic theories from the past; uses the 2017 Chinese General Social Survey (CGSS) data; comprehensively employs the least-squares method, the ordered logit regression model, and the propensity score matching (PSM) model; empirically analyzes the impact of Internet use on the life satisfaction of the elderly in China 60 years old and above and its impact mechanism; and puts forward corresponding policy suggestions for monitoring Internet use of Chinese elderly to improve their life satisfaction. The marginal contribution of this article is as follows:

- It summarizes the systematically sorted research results on the impact of Internet use by the elderly on their life satisfaction and provides literature references for scholars in further research.
- In terms of sample selection, we used the latest 2017 CGSS because it is the most representative data about China's current social situation to examine the relationship between Internet use and the subjective welfare of the Chinese elderly.
- Based on the existing research, taking into account the differences in the elderly groups, the research content also includes group heterogeneity. We conducted the comparative analyses on groups divided by age, gender, household registration, education level, health status, and personal income to further clarify the differences in life satisfaction of different groups. Besides, we elaborated and empirically tested the mechanism by which Internet use may affect their life satisfaction through the perception of social justice.
- Comparing with existing positive research in China, we found that Internet use will, on the contrary, reduce the life satisfaction of the elderly. To explain this phenomenon, we test the applicability of the existing theory to the Chinese sample.
- We put forward policy suggestions on regulating the use of the Internet for the elderly in China. Also, this provides a reference for promoting China and even the world to respond to aging actively.

This paper is organized as follows: Part II summarizes the existing studies; Part III introduces the data sources, variables selection, and the setting of the econometric model; Part IV discusses the basic results and uses the PSM model to test the robustness; Part V presents a heterogeneity analysis to discuss the differences in the effects of Internet use on life satisfaction among different older adults; Part VI presents a mechanism analysis to explore the possible mechanisms by which Internet use affects the life satisfaction of the elderly; Part VII concludes and proposes corresponding policy suggestions.

## Methods

### Data

The data used in our study is the latest released 2017 CGSS. CGSS is the earliest academic survey project in China that has national, comprehensive, and continuous features. The survey collects comprehensive information on the behavior of Chinese people, including attitude, life, and work. Also, a continuous survey could reflect Chinese society's changes. Therefore, it is the ideal dataset for cross-country comparative studies. Additional to the 2015 questionnaire, the 2017 data add modules such as life satisfaction and network society, which exactly fulfill our need for research. To better serve the research, we retain 1,344 valid observations after limiting the sample to older people aged 60 and above and removing the missing values.

### Measures

1. Dependent variable

The target variable is *life satisfaction*. Following Du and Wang (19), we obtain the variable from the question in the CGSS, “In general, are you satisfied with your living conditions?” The life satisfaction is divided into “Very dissatisfied,” “Dissatisfied,” “Neutral,” “Satisfied,” and “Very satisfied,” and assigned values from 1 to 5, with 1 being “Very dissatisfied” and 5 being “Very satisfied.”

2. Explanatory variables

The primary explanatory variable is whether people use the Internet. According to Du and Wang (19), we define the use of the Internet as the behavior that the elderly using obtain information through the Internet by devices like computers, smartphones, or smart TVs. The variable obtained from question A28 in the lifestyle module of the CGSS questionnaire, “In the past year, your frequency of Internet use (including mobile Internet) is?” The available answers are “Never,” “Rarely,” “Sometimes,” “Frequent,” “Very Frequent.” In our research, the variable *use of the Internet* is set to 0 if the answer is “Never,” and other answers are set to 1. Also, we build the variable *frequency of Internet use* to test robustness. The variable is assigned values 1, 2, 3, 4, and 5, corresponding to “Never,” “Rarely,” “Sometimes,” “Frequent,” and “Very Frequent,” respectively.

3. Control variables

Based on current research (18, 19, 63), we select the demographic characteristics (gender, age, health status, ethnic), family characteristics (number of family members), and socioeconomic characteristics (education, annual income, housing situation, political status, registered residence) of the interviewees as the control variables.

4. Mediating variables

Based on the aforementioned literature analysis, the impact of Internet use on older adults' life satisfaction is mainly through two aspects: First, Internet use may reduce the social interactions in the real life; second, Internet use distorts the perception of social fairness by expanding social comparison objects, thus reducing the life satisfaction (64). Therefore, we, respectively, selected the frequency of gathering with friends and sense of social fairness as mediating variables to analyze the influence mechanism of Internet use on life satisfaction.

Specifically, the sense of social fairness, based on the CGSS questionnaire, “In general, do you think that today's society is fair or unfair?”, the responses were reassigned into five levels from small to large, and higher numerical values represent that feel the society is fairer. Offline social interactions were characterized by friends' gatherings, and the responses to the question “How often did you gather with your friends in the past year” were reassigned with higher numerical values representing more frequent offline gatherings with friends. The specific variable descriptions are shown in Table 1.

**Table 1:** Variables.

**Variable**	**Definition**
Life satisfaction	Very dissatisfied = 1, Dissatisfied = 2, Neutral = 3, Satisfied = 4, Very satisfied = 5
Use of the Internet	Use Internet = 1, not use Internet = 0
Frequency of Internet use	Never = 1, Rarely = 2, Sometimes = 3, Frequent = 4, Very Frequent = 5
Gender	Male = 1, Female = 0
Age	Derived by survey year minus the respondent's birth year
Ethnic	Han = 1, non-Han = 0 (including all minor ethnics)
Registered residence	Non-agricultural household registration = 1 Agricultural household registration = 0
Political status	Member of the Communist Party of China (CCP) = 1; others = 0
Education	Uneducated = 0, Private school = 3, Elementary school = 6, Junior high school = 9, Senior high school or technical school = 12, College degree = 15, Bachelor's degree or above = 16
Health status	Very unhealthy = 1, unhealthy = 2, moderately healthy = 3, Healthy = 4, Very healthy = 5
Number of family members	Total number of family members
Housing situation	Owning at least one house = 1, not owning a house = 0
Annual income	Annual income in 2016, transformed by the logarithm
Sense of social fairness	Generally speaking, do you think that today's society is fair or unfair? There are five levels from low to high, and higher numerical values represent that feel the society is fairer.
Friends gatherings	In the past year, the frequency of gatherings with friends was never = 1, several times a year or less = 2, several times a month = 3, several times a week = 4, every day = 5

*Private school is a kind of folk early childhood education institution opened within the family, clan, or village in ancient Chinese society. It was a school run by private individuals in ancient and centered on Confucianism*.

### Models

1. OLS regression model

To explore whether Internet use will affect life satisfaction and determine the degree of impact, we first use the OLS model to perform a preliminary regression. The model is set as follows:

(1) $$\begin{array}{l} Satisfaction_{i}=\alpha +\beta_{1}Net_{i}+\gamma X_{i}+\varepsilon_{i} \end{array}$$

Satisfaction_*i*_ represents life satisfaction; *Net*_*i*_ represents whether to use the Internet; *X*_*i*_ is other factors that affect happiness; ε_*i*_ is the error term; β_1_ is the parameter we focus on, reflecting the direction and degree of Internet use's impact on life satisfaction.

2. Ordinal regression model

Considering that life satisfaction is typical ordinal data, we also use the ordered logit model (Ologit) for regression. The model is as follows:

(2) $$\begin{array}{l} Satisfaction_{i}=\alpha +\beta Net_{i}+\gamma X_{i}+\varepsilon_{i} \end{array}$$

Satisfaction_*i*_ represents life satisfaction of individual*i*; *Net*_*i*_ represents whether individual *i* uses the Internet; *X*_*i*_ is other factors that affect happiness; *Satisfaction*_*i*_ is the response variable, when *Satisfaction*_*i*_ lower than the critical value *C*_1_, the elderly feel very dissatisfied (*Satisfaction*_*i*_ = 1), when it is higher than *C*_1_ but lower than *C*_2_, they feel dissatisfied (*Satisfaction*_*i*_ = 2), and so on. When *Satisfaction*_*i*_ is higher than *C*_4_, the elderly feel very satisfied (*Satisfaction*_*i*_ = 5), as shown below:

(3) $$\begin{array}{l} Satisfaction_{i}=\{\begin{array}{l} 1,Satisfaction_{i}\leq C_{1}\\ 2,C_{1}<Satisfaction_{i}\leq C_{2}\\ ......\\ 5,C_{4}<Satisfaction_{i} \end{array} \end{array}$$

Assuming that ε_*i*_ follows the logistic distribution, *X*_*i*_ represents all explanatory variables, and Λ(•) represents the cumulative distribution function, *Satisfaction*_*i*_ can be expressed as:

(4) $$\begin{array}{l} \{\begin{array}{l} P\left(Satisfaction_{i}=1\right)=\Lambda \left(C_{1}-X\beta \right),Satisfaction_{i}=1\\ P\left(Satisfaction_{i}=2\right)=\Lambda \left(C_{2}-X\beta \right)-\Lambda |\left(C_{2}-X\beta \right),\\ Satisfaction_{i}=2\\ ......\\ P\left(Satisfaction_{i}=5\right)=1-\Lambda |\left(C_{4}-X\beta \right),Satisfaction_{i}=5 \end{array} \end{array}$$

The coefficients estimated by the Ologist model only give us limited information, and they do not explain the marginal effects of Internet use. Therefore, in the empirical analysis, we calculate the marginal effects of each explanatory variable, that is,

(5) $$\begin{array}{l} \frac{\partial p\left(Satisfaction_{i}=5\right)}{\partial X}=\beta \Lambda \left(C_{4}-X\beta \right) \end{array}$$

3. Propensity score matching (PSM) model

Internet use can be self-selective behavior. For example, people with better living conditions and higher self-evaluation are intended to accept and use the Internet earlier, leading to potential biases in the estimated results. Therefore, the PSM method is applied for the robustness test.

The basic idea of PSM is to reduce the observable multiple individual characteristic information to one dimensional through the probit or logit method and obtain the individual propensity score. Then match the treatment group and the control group in the sample according to the propensity score, and calculate the participant average treatment effect (average treatment effect, referred to as ATT).

In this article, the dummy variable *D*_*i*_ = [0, 1] indicates whether the elderly use the Internet, where 1 is the treatment group, representing the elderly use the Internet; 0 is the control group, representing the elderly do not use the Internet. Set the extent that the life satisfaction of the elderly is affected by Internet use as *y*_*i*_, the treatment effect of *D*_*i*_ on *y*_*i*_ is:

(6) $$\begin{array}{l} y_{i}=\{\begin{array}{cc} y_{1i}, & D_{i}=1\\ y_{0i}, & D_{i}=0 \end{array} \end{array}$$

In the above expression, *y*_1*i*_ represents the life satisfaction of the elderly who use the Internet, and *y*_0*i*_ represents the life satisfaction of the elderly who do not use the Internet. Then, the treatment effect of Internet use on life satisfaction is:

(7) $$\begin{array}{l} y_{i}=\left(1-D_{i}\right)y_{0i}+D_{i}y_{1i}=y_{0i}+\left(y_{1i}-y_{0i}\right)D_{i} \end{array}$$

Average treatment effect of participants is:

(8) $$\begin{array}{l} ATT=E\left[y_{1i}|D_{i}=1,P\left(X\right)\right]-E\left[y_{0i}|D_{i}=1,P\left(X\right)\right] \end{array}$$

4. Mediating effect model

In order to explore whether Internet use affects the elderly's life satisfaction through the sense of social fairness and friends' gathering, here in this paper, referring to the mediation effect test procedure proposed by Wen and Ye (65), Equations (8, 9) are added on the basis of Equation (1):

(1) $$\begin{array}{l} Satisfaction_{i}=\alpha +\beta_{1}Net_{i}+\gamma X_{i}+\varepsilon_{i} \end{array}$$

(9) $$\begin{array}{l} M_{i}=\alpha +\beta_{2}Net_{i}+\gamma X_{i}+\varepsilon_{i} \end{array}$$

(10) $$\begin{array}{l} Satisfaction_{i}=\alpha_{2}+\beta_{3}Net_{i}+\delta M_{i}+\gamma_{3}X_{i}+\varepsilon_{i} \end{array}$$

The coefficient β_1_ in formula (1) represents the total effect of elderly people's use of the Internet on life satisfaction;

the coefficient β_2_ in formula (8) represents the effect of volunteer service participation on the mediating variable*M*_*i*_; the coefficient δ in formula (9) is the effect of the mediating variable *M*_*i*_ on life satisfaction when controlling for Internet use, and the coefficient β_2_ represents the direct effect of Internet use on life satisfaction when controlling for the mediating variable. At this point, the mediating effect is equal to the indirect effect, that is, the product of the coefficients β_2_δ and its relationship with the direct effect and the total effect is as follows:

(11) $$\begin{array}{l} \beta_{1}=\beta_{3}+\beta_{2}\delta \end{array}$$

Among them, *M*_*i*_ is the intermediate variable. According to the mediation effect test method of Wen and Ye (65), the first step is to test the effect of using the Internet on life satisfaction, that is, to estimate the coefficient β_1_ of formula (1), if it is significant, the mediating effect should be used; otherwise, the suppressing effect should be used; the second step is to test β_2_ and δ; in turn, if all significant, the mediating effect is significant; if at least one is not significant, continue to use the Bootstrap method to test; in the third step, if β_2_ is not significant, the direct effect is not significant, indicating that there is only the mediating effect and no direct effect; if significant, it is a partial mediating effect, and if the sign of β_2_ and δ is different, it can be considered the suppressing effect. It should be noted that if there is a partial mediation effect, the effect size can be written as $\left|\frac{\beta_{2}\delta }{\beta_{1}}\right|$.

## Results

### Descriptive Analysis

Table 2 shows the descriptive statistics of the sample data. The overall status of life satisfaction of the elderly is high, with an average of 3.83. In terms of Internet use of the elderly, the majority (76.5%) never have access to the Internet, and only 23.5% had Internet activities. Among the elderly Internet users, 7.22% use the Internet very frequently, 6.92% use it relatively frequently, and 4.61% sometimes use it.

**Table 2:** Descriptive statistics.

**Variable**	**Mean/%**	**Standard deviation**	**Variable**	**Mean/%**	**Standard deviation**
*Explained variables*			Registered residence (%)		
Life satisfaction	3.83	0.83	Agricultural	60.49%	
*Explanatory Variables*			Non-agricultural	39.51%	
Internet use			CCP (%)	15.03%	
Internet user (%)	23.51%	0.42	Education (%)		
Frequency (%)			Uneducated	23.29%	
Never	76.49%		Elementary school or private school	32.36%	
Rarely	4.76%		Junior high school	24.33%	
Sometimes	4.61%		Senior high school	13.02%	
Frequent	6.92%		College degree	7%	
Very frequent	7.22%		Health	3.00	1.05
*Control variables*			Family members	2.44	1.41
Age	69.30	7.29	House owner (%)	68.60%	
Male (%)	47.62%		Family income (ln)	10.3	2.83
Ethnic (%)	93.53%				
*Mediating variables*					
Sense of social fairness	2.11	1.05			
Friends gatherings	3.27	1.03			

In terms of other features in our sample, the average age of the entire dataset is 69.3 years, of which around 47.6% are males and around 52.4% are females. Among the elderly, the majority belong to Han ethnic group; about 60.5% with agricultural registration residence; 15% with education level junior high school and above; 44.4% are in good health, with an average score of 3. The average number of household members is about 2.4; 68.6% own one or more houses.

### Simple Regression Results

In Table 3, Model 1 and Model 2 are the OLS and Ologit estimation results of the impact of Internet use on life satisfaction, respectively. Model 3 and Model 4 analyzed the relationship between the frequency of Internet use and life satisfaction.

**Table 3:** Estimated impact of Internet use on life satisfaction.

**Variable**	**Model 1**	**Model 2**	**Model 3**	**Model 4**
Internet use	−0.189^***^	−0.520^***^		
	(0.0577)	(0.151)		
Frequency of Internet use			−0.059^***^	−0.167^***^
			(0.018)	(0.049)
Age	0.0110^***^	0.030^***^	0.011^***^	0.030^***^
	(0.003)	(0.008)	(0.003)	(0.008)
Gender	−0.087^*^	−0.248^**^	−0.085^*^	−0.241^**^
	(0.047)	(0.121)	(0.047)	(0.121)
Ethnic	−0.150	−0.533^**^	−0.150	−0.536^**^
	(0.100)	(0.270)	(0.100)	(0.273)
Registered residence	0.057	0.209	0.053	0.205
	(0.052)	(0.136)	(0.052)	(0.137)
Political status	0.065	0.134	0.067	0.148
	(0.0599)	(0.164)	(0.061)	(0.164)
Education	0.015^**^	0.040^**^	0.014^**^	0.038^**^
	(0.006)	(0.016)	(0.006)	(0.016)
Health	0.195^***^	0.477^***^	0.195^***^	0.477^***^
	(0.024)	(0.065)	(0.024)	(0.064)
Flic	0.025	0.073^*^	0.024	0.073^*^
	(0.016)	(0.043)	(0.017)	(0.044)
Housing	0.071	0.133	0.073	0.140
	(0.053)	(0.133)	(0.053)	(0.133)
Annual income	0.023^**^	0.057^**^	0.023^**^	0.0563^**^
	(0.009)	(0.022)	(0.009)	(0.022)
Cons	2.235^***^		2.301^***^	
	(0.284)		(0.287)	
Cut 1		−0.246		−0.438
		(0.752)		(0.762)
Cut 2		1.557^**^		1.365^*^
		(0.723)		(0.732)
Cut 3		2.595^***^		2.402^***^
		(0.721)		(0.729)
Cut 4		5.897^***^		5.703^***^
		(0.743)		(0.751)
*N*	1,344	1,344	1,344	1,344
*R2/Pseudo-R2*	0.099	0.050	0.099	0.046

*Standard errors are reported in the parentheses*;

* *p < 0.1*,

** *p < 0.05*,

*** *p < 0.01*.

As the regression results show, first, Internet use will significantly reduce the life satisfaction of the elderly. Second, as of the frequency of Internet use, the higher the frequency of use, the more serious the deterioration of life satisfaction. Personal characteristics and socioeconomic factors also have a significant impact on the life satisfaction of the elderly. Age has a significant positive effect on life satisfaction as well as the logarithm of personal annual income. Generally, male elderly show lower life satisfaction than females. In ethnic conditions, compared with Han, ethnic minority elderly show higher happiness. Both education years and income status show a significant positive impact on life satisfaction. Health status and the number of family members also positively impact the life satisfaction of the elderly.

### Robustness Test: PSM Estimation Results

Life satisfaction varies for the elderly, which may affect they choose to use the Internet or not, so there may be a self-selection problem. Therefore, if we only use regression analysis to estimate the impact of Internet use on the life satisfaction of the elderly, the estimated results obtained may be biased. This paper further uses the PSM method to estimate the average treatment effect of participants. The group of elderly using the Internet is considered the treatment group, and the group of elderly not using the Internet is treated as the control group. Four methods are used: K-nearest neighbor matching, radius matching, kernel matching, and local linear matching. The results are shown in Table 4.

**Table 4:** The average treatment effect of Internet use on life satisfaction.

**Matching method**	**Experimental group**	**Control group**	**ATT**	**Bootstrap standard error**	***T*-value**
K-nearest neighbor matching	3.844	3.971	−0.126^*^	0.065	−1.95
Radius matching	3.844	3.996	−0.152^**^	0.058	−2.61
Kernel matching	3.844	4.005	−0.161^**^	0.059	−2.72
Locally linear matching	3.844	4.003	−0.159^*^	0.063	−2.50

***, **, and * indicate the variable's significance at the statistical level of 1, 5, and 10%, respectively; the standard error after matching is obtained by the Bootstrap with a sample size of 1,000. Standard errors are reported in column 5.

In K-nearest neighbor matching, compared with the control group, the treatment group's life satisfaction is lower by about 0.126, and the result is significant at the level of 10%. Then, we use radius matching and kernel matching to ensure accuracy. The average treatment effects obtained from the matching results are relatively similar. The treatment group's life satisfaction on average decreases by 0.15 units, which is consistent with the previous simple regression results. Therefore, we can conclude that the use of the Internet does have a significant inhibitory effect on the life satisfaction of the elderly.

## Heterogeneity Analysis

The impact of Internet use on the life satisfaction of the elderly may be affected by personal characteristics and socioeconomic factors. Based on the full sample regression referring to Jin and Zhao (18) and Du and Wang (19), we further examine the differences in the impact of Internet use on elderly life satisfaction under different factors such as age, gender, education level, urban–rural differences, health status, and income level through heterogeneity analysis.

Table 5 reports the estimation results. We can see that Internet use has a significant inhibitory effect on the life satisfaction of the elderly aged 60 to 80, especially within the range 60 to 70, but not so much for the elderly over 80 years old. From the perspective of genders, Internet use has a more substantial inhibitory effect on men's life satisfaction. Compared with women, men are more likely to overuse the Internet, reducing their life satisfaction. In terms of education level, Internet use has a significant inhibitory effect on the groups with an elementary school education level and below. Internet use has a significant inhibitory effect on the life satisfaction of the elderly with non-agricultural residence registration. However, it has no significant effect on the elderly with agricultural residence registration. Meanwhile, in terms of health level, Internet use has a significant inhibitory effect on the life satisfaction of the elderly with better health. In terms of income level, Internet use has a significant inhibitory effect on the life satisfaction of low-income elderly but has no significant effect on high-income elderly.

**Table 5:** Heterogeneity analysis of the impact of Internet use on life satisfaction.

**Variables**	**By age**			**By gender**	
	**60–70**	**71–80**	**Above 80**	**Male**	**Female**
Net	−0.171^**^	−0.245^**^	0.149	−0.206^***^	−0.167^**^
	(0.0731)	(0.112)	(0.186)	(0.079)	(0.083)
Control variables	Yes	Yes	Yes	Yes	Yes
*N*	787	399	158	640	704
*R*^2^	0.109	0.121	0.150	0.110	0.108
**Variables**	**By education level**			**By residence registration**	
	**Elementary school or below**	**High school**	**College or above**	**Urban**	**Agriculture**
Net	−0.406^***^	−0.060	−0.144	−0.208^***^	−0.123
	(0.138)	(0.071)	(0.150)	(0.072)	(0.099)
Control variables	Yes	Yes	Yes	Yes	Yes
*N*	748	502	94	531	813
*R*^2^	0.077	0.129	0.427	0.117	0.088
**Variables**	**By health status**			**By income level**	
	**Unhealthy**	**Moderately healthy**	**Healthy**	**Low income**	**High income**
Net	−0.261	−0.169^*^	−0.193^**^	−0.155^***^	−0.377
	(0.164)	(0.0967)	(0.0752)	(0.058)	(0.243)
Control variables	Yes	Yes	Yes	Yes	Yes
*N*	444	445	455	892	452
*R*^2^	0.055	0.052	0.133	0.109	0.075

*Standard errors are reported in the parentheses*;

* *p < 0.1*,

** *p < 0.05*,

*** *p < 0.01*.

## Mechanism Analysis

Internet use has a significant negative impact on older adults' life satisfaction, but the channels through which affects older adults' life satisfaction require further analysis. The aforementioned literature analysis found that the negative effect of Internet use on older adults' life satisfaction is mainly through two aspects: First, Internet use may reduce offline social interactions; second, Internet use affects perceptions of social fairness and life satisfaction by expanding the social comparison target (64). Based on the two possible channels of the negative effects and the availability of data, in the first channel, we selected the frequency of gathering with friends as a mediating variable to characterize social interactions, and in the second channel, we selected the sense of social fairness as a mediating variable to characterize the perception of fairness in socioeconomic status, to analyze the possible influence mechanisms of Internet use on life satisfaction.

The specific method uses the method of Wen et al. (66) to test the regression coefficients in turn. First, use the Internet use to regress the self-evaluation of the intermediary variables, and then do the regression analysis of the benchmark model with the addition of mediating variables. Models 5 and 7 are the regression results of Internet use on mediating variables, and models 6 and 8 are the regression results of the benchmark model after adding the mediating variables. The specific regression results are shown in Table 6. By observing the table, Internet use only has a negative impact on life satisfaction through social fairness. However, the use of the Internet has increased the number of offline gatherings of friends, but the increase in the number of gatherings of friends has not significantly increased the life satisfaction of residents.

**Table 6:** Estimation results of mediating effects.

	**Model 5**	**Model 6**	**Model 7**	**Model 8**
Variables	Fairness	Satisfy	Friends gatherings	Satisfy
Net	−0.262^***^	−0.129^**^	0.351^***^	−0.187^***^
	(0.0785)	(0.0568)	(0.073)	(0.058)
Fairness		0.229^***^		
		(0.0252)		
Friends gatherings				−0.005
				(0.0227)
Control variables	Yes	Yes	Yes	Yes
*N*	1,336	1,336	1,343	1,343
*R*^2^	0.048	0.176	0.062	0.099
*Sobel Z test*	−0.060^***^(0.019)	−0.002(0.008)		

*Social fairness perception is divided into five levels: The higher the level, the more you feel you are treated fairly; depression is divided into five levels: The higher the level, the higher the level of depression; friends gathering here refers specifically to the frequency of gathering; there are five levels in the questionnaire: The higher the level, the more frequency. Standard errors are reported in the parentheses*;

* *p < 0.1*,

** *p < 0.05*,

*** *p < 0.01*.

## Discussion

The above results supported that the use of the Internet does have a significant inhibitory effect on the life satisfaction of the elderly. But some Chinese scholars (18, 19, 63) found that Internet use can promote the life satisfaction of the elderly, which is different from the above results. But their studies are based on the 2015 CLASS and 2013 CSS data. The Internet would occupy time for social interaction, resulting in less communication with family members, fewer opportunities for face-to-face communication between people, and increasing isolation between people. It is harmful to maintain personal and social relationships, thereby increasing inner loneliness and reducing life satisfaction (39–41). Furthermore, Gilleard et al. (42) and Hage et al. (43) believed that the use of the Internet will cause the elderly to reduce their valid social participation and weaken the elderly's sense of belonging to the community, negatively impact on the elderly's life satisfaction. Matsuba (44), Stepanikova et al. (45), Odaci and Elik (46), Azher et al. (47), Noh and Kim (48), Sami et al. (49), and Kitazawa et al. (50) found that excessive use of the Internet can make people addicted, produce loneliness, and affect mental health and life satisfaction.

Meanwhile, the relationship between the Internet use and life satisfaction is affected by education level, urban–rural distribution, and income level. From the perspective of education level, after being exposed to the Internet, groups with lower education levels are more likely to become addicted due to the low opportunity cost of addiction, which would be harmful to their mental health (67). For the difference caused by income level, one possible explanation is the gap between reality and imagination. Ideal lifestyle and all the beautiful things happening worldwide are easily exposed to the elderly by using the Internet. Higher quality of life usually requires a higher material basis. However, low-income elderly groups cannot meet their increasing material needs due to their low incomes, resulting in negative emotions such as dissatisfaction with reality and reducing life satisfaction (54).

Using the Internet will reduce the elderly's sense of fairness, which leads to the decline of life satisfaction. The Internet use will increase access to information for the elderly, allowing them to easily compare online with others from any country and background. Comparing behavior based on this will bring a sense of psychological loss and relative deprivation, and reduce people's judgment on the state of happiness in life. Clark and Oswald (58) found that through the use of the Internet, people can easily see the lives of others and compare their lives with others, thereby producing a comparison effect and reducing happiness. Internet users compare their lives with others, which may reduce life satisfaction (60). Lee (61) also found that the comparison of social networking sites tends to cause individuals to produce non-adaptive perceptions or feelings that “oneself is not as good as others” and “others are happier.” The elderly has obtained more extensive and comprehensive information through the use of the Internet, with the onslaught of various information, the elderly have more social comparisons, which has led to a decline in life satisfaction.

## Conclusion

With the rapid development of the Internet, the relationship between Internet use and the life satisfaction of the elderly has aroused widespread concern in society. This paper uses the latest 2017 China General Social Survey to investigate the impact of Internet use on the life satisfaction of the elderly. The results show that (1) Internet use will significantly reduce the elderly's life satisfaction. A higher frequency of using the Internet usually leads to lower life satisfaction. To reduce endogenous bias, we use the PSM model to conduct a robustness analysis, which further validates this observation. (2) Considering the different elderly groups, we conduct heterogeneity analysis on age, gender, household registration, education level, health status, income levels, and find that Internet use has a significant inhibitory effect on elderly groups with the characteristic of males, middle-to-low-age elderly, low-income elderly, non-agricultural registered, lower educational or better health. Based on the above empirical results, we give policy suggestions on how to prevent negative impact brought by Internet use on the life satisfaction of the elderly as follows:

(1) Improve the elderly's attitudes toward Internet use. Although the Internet usage in China is increasing day by day, many of the elderly still lack a reasonable understanding of the Internet. Especially, for the group of the elderly, due to the lack of rational attitude, they fail to accept the convenience brought by the Internet, and even some mental diseases are caused by overusing or abusing the Internet.

(2) Pay attention to the sense of fairness of the elderly to improve life satisfaction. The above analysis showed that the decline of the sense of fairness of the elderly will reduce life satisfaction. The reduction of the sense of fairness comes from the behavior of comparing with others in the process of using the Internet. The elderly should try to be optimistic in the process of using the Internet, reduce the comparison between themselves and others on the Internet, and accept the information presented on the Internet optimistically.

(3) Guide the elderly to reduce the time of using the Internet. According to our results, the more frequently you use the Internet, the more likely it will be harmful to the elderly's life satisfaction. So it's necessary for the elderly to control their time on using the Internet, although the Internet is everywhere. Overly dependence on the Internet will reduce face-to-face communication time, seriously affecting their daily life, and interpersonal communication. Therefore, it is necessary to guide the elderly on how to use the Internet rationally, help them participate in more practical activities, and reduce the negative impact of the Internet on life satisfaction.

## Limitations

Although this paper comprehensively analyzed the relationship between the Internet use and life satisfaction, there are certain limitations: First, due to the limitations of publicly available survey data, the selection of samples may not be comprehensive, and an important mediator is a content seen/browsed on the Internet that cannot be added to the model; second, the analysis of endogenous issues needs to be further solved; third, the mechanism of how the Internet use affects life satisfaction for the elderly needs to be further explored. These shortcomings will provide research directions for future in-depth research.

## Data Availability Statement

The raw data supporting the conclusions of this article will be made available by the authors, without undue reservation.

## Author Contributions

X-yL and H-lY conceived this research. LX was responsible for the methodology. SZ conducted software analyses. S-qZ and S-mT conducted necessary validations. Y-yW conducted a formal analysis and managed the investigation. LX and X-yL gathered resources, curated all data, wrote/prepared the original draft, and were responsible for project administration. X-dZ and H-lY reviewed and edited the manuscript, were responsible for visualization, supervised the project, and acquired funding. All authors contributed to the article and approved the submitted version.

## Conflict of Interest

The authors declare that the research was conducted in the absence of any commercial or financial relationships that could be construed as a potential conflict of interest.
